# Predictive correlation of optimum phase change material for thermal energy storage

**DOI:** 10.1038/s44172-026-00655-y

**Published:** 2026-04-09

**Authors:** Ayushman Singh, Srikanth Rangarajan, Bahgat Sammakia

**Affiliations:** 1 Department of Mechanical Engineering, Binghamton University, Binghamton, NY USA; 2 School of Systems Science and Industrial Engineering, Binghamton University, Binghamton, NY USA

**Keywords:** Energy modelling, Mechanical engineering

## Abstract

Organic phase change material and high thermal conductivity filler composites are effective in thermal energy storage, but their performance can be highly dependent on the volume fraction of the phase change material/filler. In these composites, energy density (energy storage capacity of the composite) can be enhanced by increasing the phase change material volume fraction; in contrast, power density (rate at which the energy is accessed) can be improved by increasing the filler volume fraction. However, both characteristics cannot be increased simultaneously; therefore, the optimum volume fraction is crucial for maximizing the composite’s thermal performance. In this work, we leverage the analogy of the thermal energy storage in phase change material with electrochemical energy storage in batteries through the Ragone framework to determine the optimum design. Furthermore, this paper proposes relationships for the optimum volume fraction as a function of thermal load, geometry, and cutoff temperature during the melting process. The proposed correlations provide a practical tool for designing and optimizing organic phase change material-filler composites and reduce reliance on computationally intensive numerical or experimental trials.

## Abstract (web summary)

Ayushman Singh and colleagues propose predictive correlations for optimizing organic phase change material composites by leveraging Ragone framework. The results can aid in quick and accurate design decisions for phase change material based thermal systems.

## Introduction

Thermal energy storage (TES) systems for passive transient thermal management have been a focus of attention for decades, with applications in building air conditioning^1^, thermal management of batteries^2^, and electronic devices^3–5^. The employment of phase change materials (PCMs) in TES systems is principally due to the energy storage capability of the PCMs in the form of latent heat of fusion during the transition from solid to liquid phase. High latent heat of fusion of PCMs delivers high energy density, the amount of energy stored per unit mass or volume. The other characteristic is power density, defined as the rate at which this energy is accessed by the PCM. During the transition phase, PCMs aid in maintaining a temperature near the melting point or phase transition temperature. In electronic devices, PCM can be used as a thermal buffer to mitigate temperature rise beyond a cutoff or threshold temperature during intermittent heat loads. Different categories of PCMs have been used to safely operate electronic chips, including organic alkanes, inorganic salt hydrates, low-melting-temperature metals, and eutectic alloys. These categories of PCMs have some advantages and disadvantages in terms of their thermophysical properties. For instance, organic PCMs, such as paraffin wax, have high gravimetric latent heat (240 J g^−1^) but low intrinsic thermal conductivity (0.2 W m^−1^ K^−1^). In contrast, metal (gallium) or eutectic alloys (field’s metal) have a high thermal conductivity of 20–30 W m^−1^ K^−1^ but low gravimetric latent heat (82.3 J g^−1^)^6^. The latent heat of fusion dictates the energy density available, whereas the thermal conductivity dictates the rate at which the available energy density can be accessed, i.e., power density (W m^−3^).

Organic PCMs often have low power density due to their low thermal conductivity. This results in a portion of the PCM’s available energy density being inaccessible due to the temperature threshold. Ideally, consuming most of the available energy of the PCM should be desirable before the threshold temperature is reached for the device’s safety during heat spikes. The power density can be increased by adding highly thermal-conducting fillers or matrices; however, under a volume or mass constraint, the available energy density is compromised as some portion of the PCM needs to be removed to add the filler or matrix material. This leads to a competing trade-off between energy density and power density, where one characteristic deteriorates in an attempt to improve the other one. Ideally, both energy and power density are desired to be higher under the volume or mass constraints.

The quantification of the performance of the PCM TES systems has often been done using metrics, such as the time to reach a set point or cut-off temperature or the temperature at the end of the heating duration^7,8^. However, a unified system is required to define the performance metric that could be expressed in terms of variables that affect the performance. This development can be expensive with experimentation or high-fidelity numerical simulations; therefore, a simplified numerical model can be a helpful tool for comparing different cases. Cooling capacity figure of merit (FoM) has been proposed in several studies that aim to quantify the balance between the energy and power density for TES systems^9,10^. However, these figures of merit are based mainly on the inherent material properties of the PCM or the effective properties of the PCM-filler composite. This FoM is an excellent tool to estimate the balance between the thermal conductivity and the heat storage capability of a material, but does not take into account the effect of the magnitude of the boundary conditions, cutoff temperature, or the geometry. Several attempts have been made to modify this FoM in order to include the effects of the factors that could affect the performance of the PCM-based thermal buffer. For instance, modifying the conventional FoM by adding a performance factor to account for the effect of geometry on thermal performance^11^. Additionally, several efforts have also been made to analytically determine the power-energy trade-off and leverage it to understand the effects of these operating conditions^12–14^. One of such study proposed a theoretical framework to design and optimize cylindrical composites with three figures of merit: minimization of temperature rise, maximization of the effective volumetric heat capacity, and maximization of the effective heat capacity based on mass^13^. The work demonstrates that optimum volume fraction shifts depending on objective and thermal load, and validates this with experiments. Same researchers in another study discussed the limits/roles of the overall geometry of the system and thermal load being buffered, on the critical pitch^14^. Their work focused on lamellar (layered) PCM composites and provides an efficient framework to explore how volume fractions, orientation, and length scales affect performance. They concluded that the optimum volume fraction of high thermal conductivity material within the composite occurs when the rate-limited thermal storage and capacity-limited thermal storage are set equal.

There are several experimental^7,15,16^ and numerical^17–20^ studies performed for the optimization of the PCM TES systems. Though these literatures share meaningful insights about PCM TES, there are some limitations in these studies. Firstly, most of the experimental studies are performed over a limited range of operating conditions, especially at low heat flux, covering a narrow set of applications. Secondly, it is very challenging to experimentally test a wide range of design cases in terms of different volume fractions of PCM because of manufacturing and economic limitations. In these attempts, whether it is experimental or numerical, the performance metrics, such as maximizing the operation time or minimizing the temperature at the end of heating duration are generally used as the objective functions. These performance metrics are often not applicable to a wide range of operating conditions and are hard to generalize on a single framework^21^. Therefore, there is a need to obtain a framework to understand the behavior of the PCM heat sink under varying conditions of thermal load, cut-off temperature, and geometry (aspect ratio) to determine the ideal configuration that is best suited for the given operating conditions. A follow-up to this challenge is to generate a simplified numerical model based on reasonable assumptions that could lead to generating a unified framework applicable to a wide range of applications. Some attempts have been made where simplified models have been utilized to understand the balance between the energy storage and heat conduction in the PCM TES systems. And some of these studies also utilized the Ragone framework along with the help simplified model to demonstrate the effect of different operating conditions, such as thermal boundary, geometry, and threshold temperature, using Ragone plots^21–25^.

The analogy of TES in PCM and electrochemical energy storage in batteries based on Ragone framework has been earlier utilized for TES heat exchangers^22,23,26^, electronic devices^24,27,28^, and battery thermal management^29^. For example, one of the studies implemented the thermal Ragone framework to understand the effect of several operating conditions on a single energy-power plot (Ragone plot) that could aid in making design decisions^22^. The same framework has also been used to design and optimize the finned tube PCM heat exchangers with validated simplified numerical model against experimental tests, addressing the volumetric and gravimetric power-energy tradeoffs^23^. In another study, power-energy tradeoff was employed on the Ragone framework to develop design rules and performance limits for conductive–capacitive PCM composites under transient heating^27^. And the authors proposed a physics-guided (Stefan-Neumann model) analysis that maps regimes where PCM composites can outperform even very high thermal conductivity materials in transient thermal buffering and articulates limits/rules for rational composite design. This framework provides a normalized metric for comparing charge–discharge behavior across different storage systems and has been successfully applied to interpret thermal charging and discharging dynamics.

While these studies have leveraged the applicability of the Ragone framework for TES systems and were useful in understanding the energy-power tradeoffs and how different operating conditions affect the performance. However, the Ragone framework was used only as a tool to compare and characterize the energy and power density of different TES systems, whereas this framework can also be used as a tool to optimize the design under different combinations of operating conditions. For example, a study considered the thermal Ragone framework to optimize planar TES heat exchanger designs with PCM and high thermal conductivity additives. Their work focused on TES heat-exchanger design to maximize effective energy density and minimize the cost^21^. However, any direct relationship in the form of correlations between the optimized design and the combination of operating conditions (design parameters) was not obtained.

In this paper, we have investigated the effects of operating conditions, such as thermal boundary, cutoff condition, and geometry, through the Ragone framework. Then, we leveraged this framework as an optimization tool to develop correlations that aid in determining an optimum composition of high thermal conductivity fillers and organic PCM as a function of the operating conditions for Cartesian and cylindrical coordinate systems.

To achieve this, we first employ the Ragone framework—based on the analogy between electrochemical and TES systems—to understand the impact of various operating conditions on the PCM TES. The data for the Ragone framework are obtained from a numerical model that has been benchmarked against experimental results. Subsequently, the Ragone framework is implemented to establish relationships between the optimal design of organic PCM-filler composite and the operating conditions.

## Electrochemical and thermal energy storage analogy

**Fig. 1:**
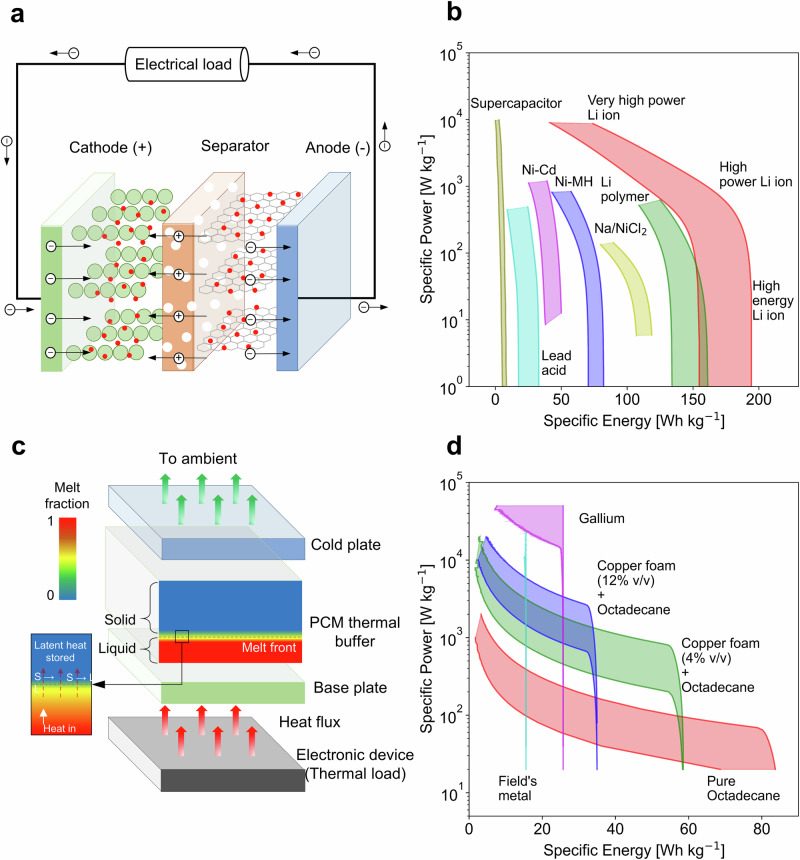
Analogy of electrochemical and thermal energy storage system. **a** Schematic showing electrochemical energy discharge in a Li-ion battery, **b** Ragone plot showing specific power vs specific energy for different batteries, **c** schematic of a phase change material thermal buffer device for an electronic module, **d** thermal Ragone plot for different phase change material thermal energy storage materials. Note: data used to generate (**b**) are taken from literature^33,34^, whereas (**d**) is generated using the present numerical model.

The energy and power densities are predominantly used in electrochemical energy storage systems, often depicted as Ragone plots, originally developed by Ragone^30^ for characterization and comparison of electrochemical energy storage systems. In the electrochemical energy storage systems, electrical energy stored during charging is discharged to an electrical load as depicted in Fig. 1a.

Figure 1b shows a Ragone plot of various electrochemical energy storage systems. It can be seen that a supercapacitor, for example, has very high specific power (W kg^−1^) but low specific energy (W h kg^−1^), whereas a high-energy Li-ion battery has high specific energy but low specific power. Hence, a supercapacitor can discharge an electrical load rapidly; however, the discharge time will be shorter as the energy density is lower. In contrast, high-energy Li-ion batteries can offer longer operation time, though at low power delivery to the load. In addition, for a given Li ion cell, the addition of conductive material, such as graphene coating on the electrode, may improve the power density; however, the active material must be sacrificed to achieve the same volume or mass.

Similarly, the latent thermal energy available in the PCM can be utilized to buffer the thermal load from electronic devices, as shown in Fig. 1c. TES systems with PCM can also be characterized based on the energy and power density, analogous to the electrochemical energy storage^22,27,31^. The thermophysical properties of the PCM that are important here are the latent heat of fusion and thermal conductivity. The latent heat dictates the energy density, and thermal conductivity dictates the power density. For example, a pure organic PCM has extremely low thermal conductivity and high latent heat per unit mass, whereas a metallic PCM, gallium, has a hundred times higher thermal conductivity than octadecane; however, latent heat per unit mass is one-third. This can be seen in Fig. 1d, where pure octadecane shows high specific energy and low specific power, whereas gallium and Field’s metal are in the low specific energy and high specific power region. Additionally, as metal fillers (copper in this example) are added to PCM, specific energy is reduced as PCM volume is sacrificed to accommodate the space for filler, and specific power is increased due to enhanced thermal conductivity of the composite. As more volume of copper filler is added, there is a greater reduction in the PCM volume, hence low specific energy, and more enhancement in the thermal conductivity, hence higher specific power.

An electrochemical cell’s Ragone plot can be produced if we are aware of the specific energy values under various specific power conditions. The specific energy’s departure from the ideal value (maximum discharge capacity) can be seen through experimental or numerical analysis. Alongside the Ragone plot, the discharge curve, also referred to as the rate capability curve, is another important characteristic curve of a cell^22,23^. The discharge curve depicts the change in cell voltage with depth of discharge (DOD) or discharge capacity. Here, DOD represents the fraction of the maximum discharge capacity delivered to the load. In the discharge curve, the threshold cell voltage below which the electrical load is unable to discharge the cell is known as the cutoff voltage (${V}_{{{\rm{c}}}}$). The DOD or discharge capacity at the cutoff condition may dictate the specific energy discharged from the cell. In contrast, the rate of discharge, also known as the *C* rate to the electrical load, may dictate the specific power. *C* rate is the ratio of discharge current to maximum discharge capacity of the cell, and a *C* rate of 1 (or 1*C*) refers to the discharge rate that can fully discharge the cell in 1 h^32^.

**Fig. 2:**
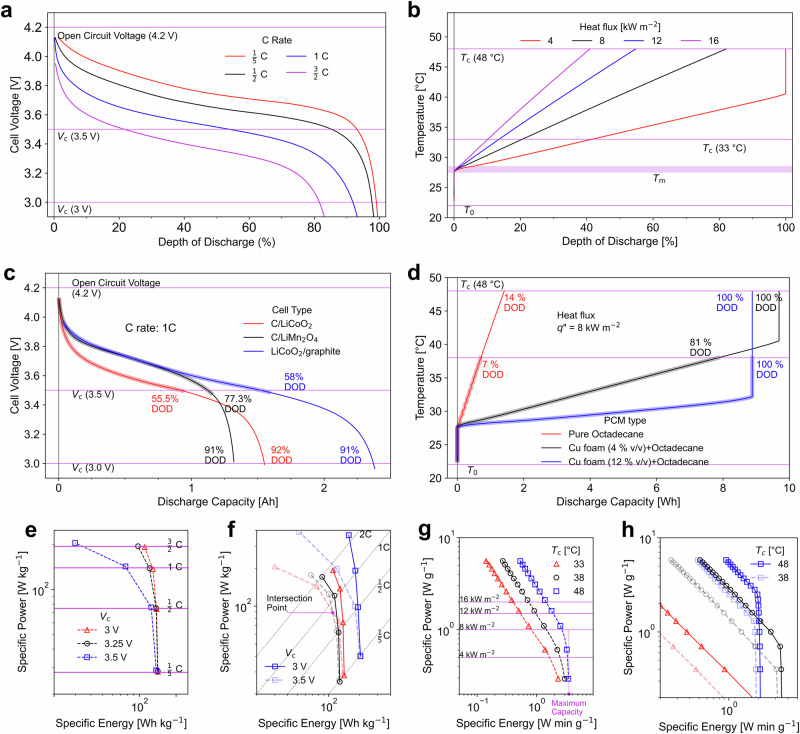
Rate capability (discharge curves) and Ragone plots for electrochemical and thermal energy storage systems. **a** Discharge curve of C/LiCoO_2_ lithium ion cell at different *C* rates, **b** discharge curve of copper foam (4% v/v) with octadecane at different heat flux, **c** discharge curve of C/LiCoO_2_, C/LiMn_2_O_4_, and LiCoO_2_/graphite lithium ion cells at *C* rate of 1, **d** discharge curve of pure octadecane, copper foam (4% v/v) with octadecane, copper foam (12% v/v) with octadecane at heat flux of 8 kW m^−2^. **e** Ragone plot based on (**a**), **f** Ragone plot based on (**c**), **g** thermal Ragone plot based on (**b**), **h** thermal Ragone plot based on (**d**). Note: data used for generating (**a**, **c**, **e**, **f**) are taken from the handbook of batteries^32,35^, whereas data from the present model are used to generate (**b**, **d**, **g**, **h**).

Figure 2a, e shows the discharge curves and corresponding Ragone plot, respectively, for the LiCoO_2_ cell at different *C* rates and cutoff voltages. As the *C* rate increases, a cell experiences a reduction in the DOD at a given cutoff voltage condition. This is primarily caused by the fact that when the current flow through the cell increases, the voltage drop across the cell’s internal resistance increases. A large voltage drop across the internal resistance causes a lower available voltage at the battery terminals. The low specific power points in the Ragone plot represent a low *C* rate, as shown in Fig. 2e, where at 1/2*C* and 1/5*C*, most of the discharge capacity is being utilized from the cell. However, as the *C* rate is increased, leading to an increase in the specific power, the specific energy deviates from the maximum discharge capacity. If the cutoff voltage is lower, there is less deviation from the maximum value.

Similarly, a phase change material thermal buffer can also be characterized based on the discharge curve and thermal Ragone plot. In the case of the transient thermal buffering of the power spike in an electronic device, the transient temperature and the maximum allowable temperature or cutoff temperature (${T}_{{{\rm{c}}}}$) at the heat-generating device can be considered analogous to the cell voltage and cutoff voltage in a cell, respectively. Figure 2b, g depicts the discharge curve and the thermal Ragone curves, respectively, for a composite of organic PCM (n-octadecane) and 96% porosity copper foam at different heat fluxes. In this case, the thermal energy stored in the composite is obtained until a cutoff temperature is reached. The heat flux dictates the specific power, and the energy stored dictates the specific energy. At lower heat flux, the DOD is 100% when the cutoff temperature is 48 °C. However, the DOD decreases as the heat flux increases, leading to a deviation from the maximum specific energy.

To compare the behavior of different cells, discharge curves and Ragone plots are shown for three different Li-ion cells in Fig. 2c, f, respectively. Please note that these cells have similar mass and volume and differ mainly in the active and anode materials, leading to different discharge curve behavior and maximum discharge capacity. Figure 2d, h shows the discharge curve and thermal Ragone curves, respectively, for pure octadecane, octadecane in 96% porosity copper foam, and octadecane in 88% porosity copper foam at a fixed total volume. Adding the metal foam can increase the discharge capacity at a given cutoff temperature condition. However, if the cutoff temperature is reduced, the need for thermal conductivity increases.

Overall, this shows that the TES in the PCM thermal buffer and the electrochemical energy storage in cells are analogous to each other. Furthermore, we illustrate how the specific power and specific energy curves vary depending on the material, thermal boundary, and cutoff temperature. The variables that impact the thermal Ragone plots and the process for generalizing them are covered in detail in the following section.

## Factors affecting the thermal Ragone plots

The thermal boundary condition or heat flux supplied on the heated side dictates the specific power (gravimetric) or power density (volumetric). The energy stored in the material until the cutoff condition dictates the specific energy or energy density. These are the two characteristics of the thermal Ragone plot.

**Fig. 3:**
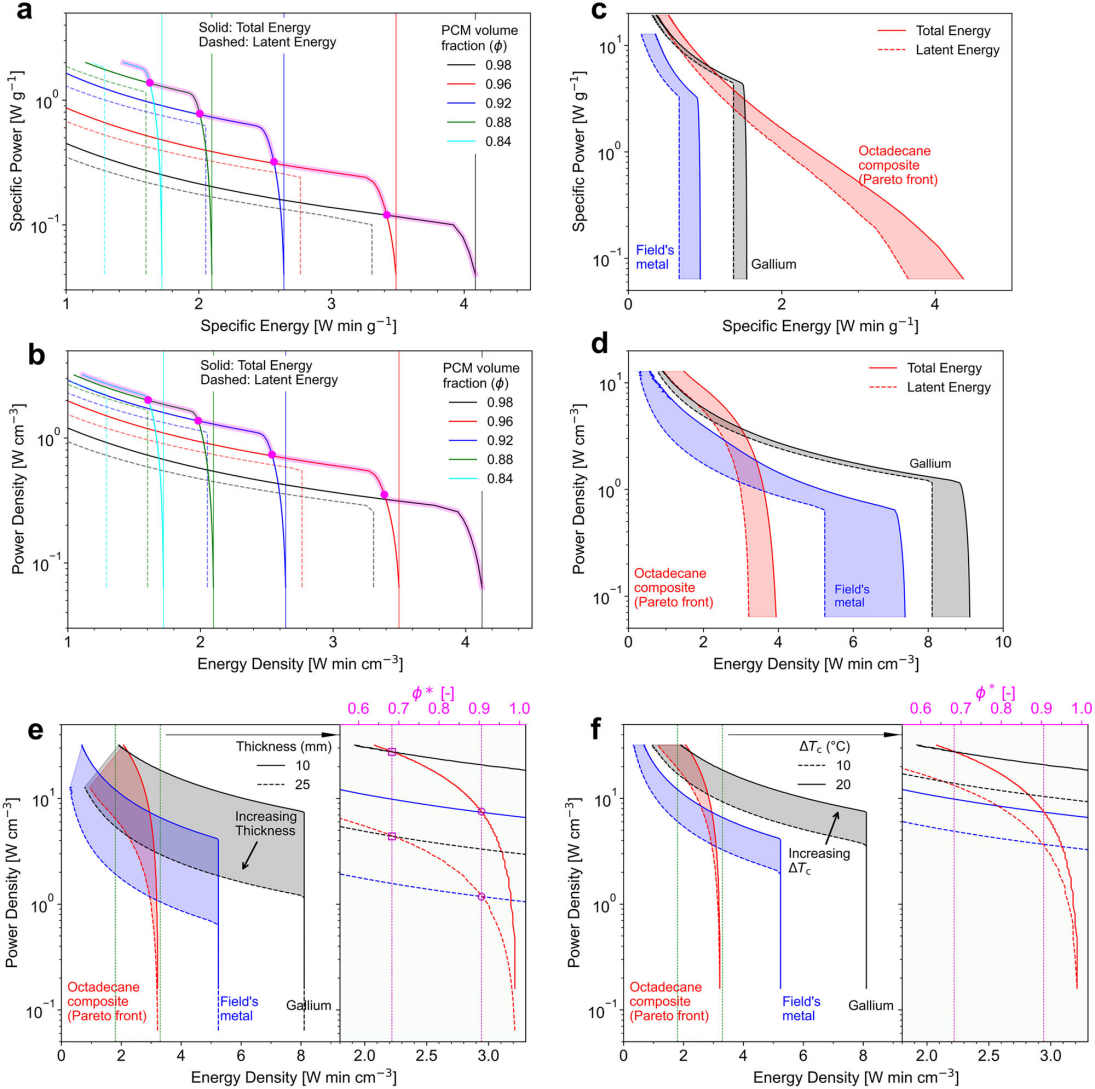
Effects of operating conditions on energy and power density for different PCMs. **a** Gravimetric and **b** volumetric thermal Ragone plots comparing different PCM volume percentages for copper and octadecane composite. Curves outlined in magenta color indicate the Pareto front of maximum specific energy or energy density, **c** gravimetric and **d** volumetric thermal Ragone plots comparing Pareto front for octadecane-copper composite with pure gallium and field’s metal, **e** effect of thickness or aspect ratio, **f** effect of cutoff temperature. Note: these plots are generated from the numerical model used in the present study.

If high thermal conductivity fillers are added to the PCM under a constrained total mass, specific energy can be increased at a specific power, even though the maximum energy storage capacity has been compromised. Figure 3a, b shows the gravimetric and volumetric thermal Ragone plots, respectively, for five different PCM volume fractions in the composite of PCM and copper fillers. The vertical lines represent the maximum energy storage capacity. As the specific power is increased, the ability of the material to store the maximum energy available is reduced. Furthermore, there is a drastic reduction in the specific energy when specific power is increased beyond a particular value (shown as the knee point^25^). At any PCM volume, the respective maximum energy storage capacity was achieved at low power. However, the knee point occurred at a higher specific power as the copper filler percentage was increased. This led to different thermal Ragone curves intersecting with each other near the knee points. These intersection points are shown as circular magenta-colored markers in Fig. 3a, b. Therefore, a Pareto front (outlined in magenta) of the maximum energy density points can be obtained.

We can also see the comparison of the octadecane-copper composite Pareto front with pure gallium (a metallic PCM) and pure field’s metal (a low melting point eutectic alloy) on the gravimetric (Fig. 3c) and volumetric (Fig. 3d) basis. Gallium and field’s metal are not used here with any thermal conductivity enhancement filler, as they inherently possess high thermal conductivity, at least 100 times higher than pure organic PCM. The shaded region represents the sensible energy storage in the PCM. In general, latent energy storage accounts for more than 80% of the total energy storage.

Please take note that Fig. 3a–d is produced for a specific buffer thickness (25 mm) and cutoff temperature (48 °C) using octadecane as the organic PCM and copper as the filler. One of the factors that could cause the specific energy to deviate from the ideal value is the cutoff temperature, as mentioned previously and illustrated in Fig. 2. Additionally, we can also say that the specific energy or energy density may vary depending on the thickness. For example, we want the PCM to be more responsive to melting before the cutoff condition is reached if the thickness is increased or the cutoff temperature is set closer to the PCM’s melting temperature. To improve the thermal conductivity, more filler volume can be added. However, adding too much filler can reduce the maximum energy storage capacity. As a result, there ought to be an optimum PCM volume fraction (${\phi }^{* }$) where a given power density can yield the highest energy density. As seen in Fig. 3e, f, respectively, results were produced for an additional thickness of 10 mm and cutoff temperature of 38 °C to comprehend how these curves would change with these variables. The thermal Ragone curves for gallium, field’s metal, and octadecane-copper composite (Pareto front) shift towards lower energy and power density as the thickness increases or the cutoff temperature decreases. The thermal Ragone curves for the octadecane-copper composite are magnified for better visibility and shown on the right side. Please note that the Pareto front of octadecane-copper carries the information of the optimum PCM volume fraction, which is represented on the secondary x-axis. It can be concluded from this section that the optimum composition of high thermal conductivity fillers in the organic PCM depends on the aspect ratio, heat flux, and cutoff temperature. For a generalized balance of energy and power density for the optimized design, a comprehensive dataset is needed. This can be accomplished either from experimental tests or numerical models. However, experiments are expensive and not feasible, especially when a large number of test cases are required. Here, numerical models can aid in generating a large number of cases to generate data to utilize the thermal Ragone framework and hence to reach a generalized solution to optimum design. In the next section, we will look at the numerical models that could help achieve the optimized design.

## Methods

### Numerical models

One way to simulate the melting process in the PCM-filler composite is to capture the energy transport in the fins and PCM separately, along with the convection effect due to buoyancy in the liquid PCM. This model is expressed here as a high-fidelity model. The other model is a low-fidelity model that solves a single one-dimensional energy equation, assuming a homogeneous medium of PCM and fins with the effective thermophysical properties. As the low-fidelity model is based on the effective medium, the effect of fin thickness and gap width cannot be taken into account. In contrast, the high-fidelity captures the effect of the fin geometry. To understand the efficacy of both the modeling approaches, experiments were performed for three different fin designs and at heat fluxes in the range of 8–120 kW m^−2^. More details on high and low fidelity models can be obtained in the supplementary document (Supplementary Notes 1 and 2, respectively). As these numerical models are the data source for the thermal Ragone framework, we need to ensure that they are validated with experimental findings within a wide range of aspect ratios, heat flux, and cutoff temperatures.

### Experimental test

**Fig. 4:**
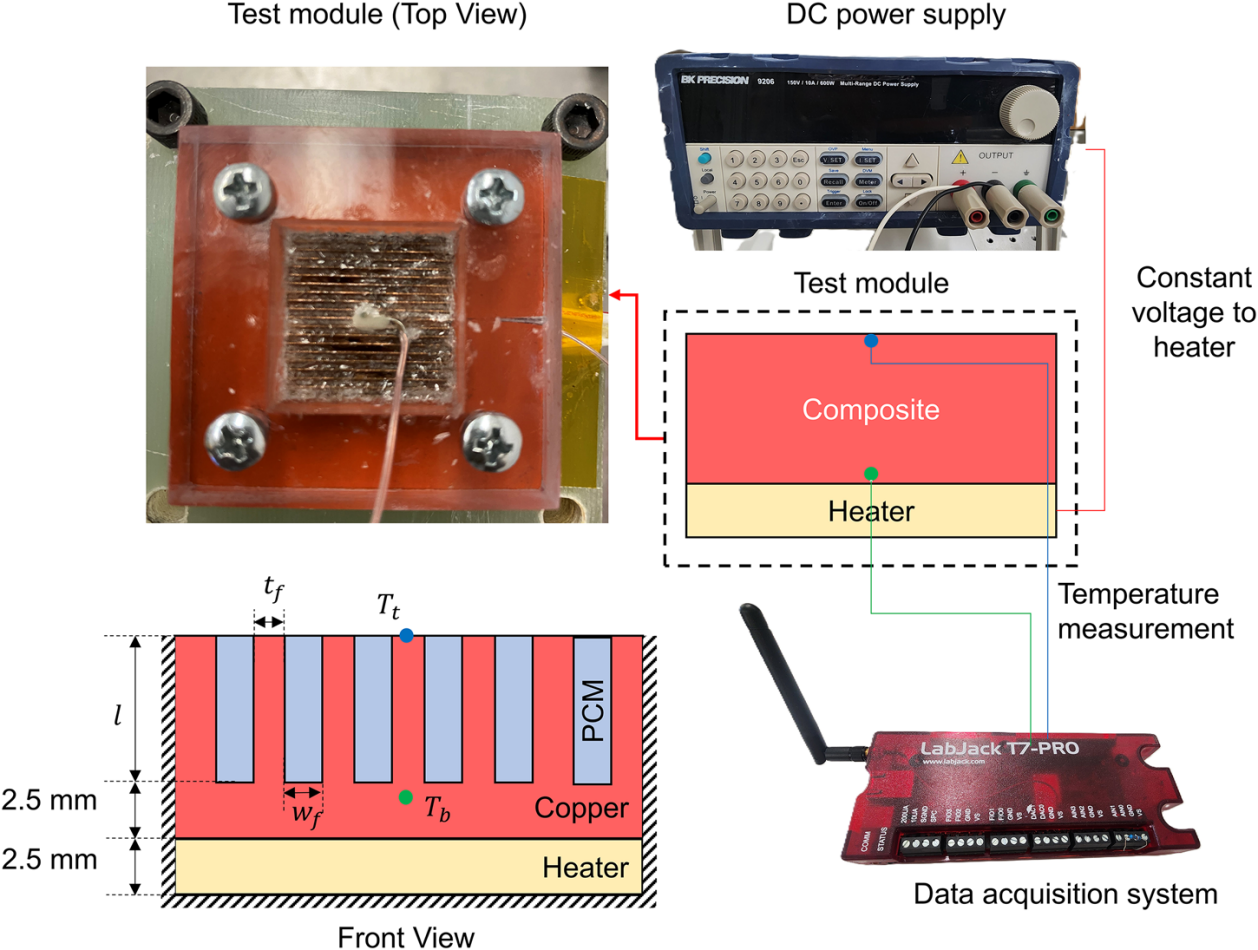
Experimental test setup showing test module, power supply unit and data acquisition system*.*

The experimental test rig used in this study, as shown in Fig. 4, consists of a test module, a data logger (LabJack T7 Pro), and a DC power supply unit (Keysight E3634A). The test module consists of a PCM embedded in copper fins and a heater (Watlow). The test module is insulated on the sides and bottom, and open to the air at the top, allowing for natural convection. Multiple T-type thermocouples (omega) with an uncertainty of ± 0.5 °C were used to measure the temperature at the bottom and top of the thermal buffer, denoted as ${T}_{{{\rm{b}}}}$ and ${T}_{{{\rm{t}}}}$, respectively. For the transient experimental test, a constant voltage is supplied from the power supply unit to the heater. At the desired constant heat flux supplied from the heater to the PCM thermal buffer, transient temperature data are recorded at the bottom and top of the thermal buffer until the bottom temperature reaches a threshold or cut-off temperature.

Transient experiments were performed for three copper finned thermal buffer designs (different PCM volume fractions) with a thickness of 5 and 25 mm. Three design cases were manufactured with the fin gap widths (${w}_{{{\rm{f}}}}$) of 0.25, 0.5, and 1 mm, with the fixed fin thickness (${t}_{{{\rm{f}}}}$) of 0.25 mm. This leads to three thermal buffer designs with different volume fractions of PCM. The volume fraction and fin geometry details of the three cases can be found in the supplementary document (Supplementary Table 1). The experimental tests were performed with octadecane (melting point, ${T}_{{{\rm{m}}}}$ = 28 °C) as PCM in the heat flux range of 8–120 kW m^−2^, varied at steps of 8 kW m^−2^. The material properties of octadecane, along with the other PCMs used in this study, are mentioned in the supplementary document (Supplementary Table 2).

### Validation of models with experiments

**Fig. 5:**
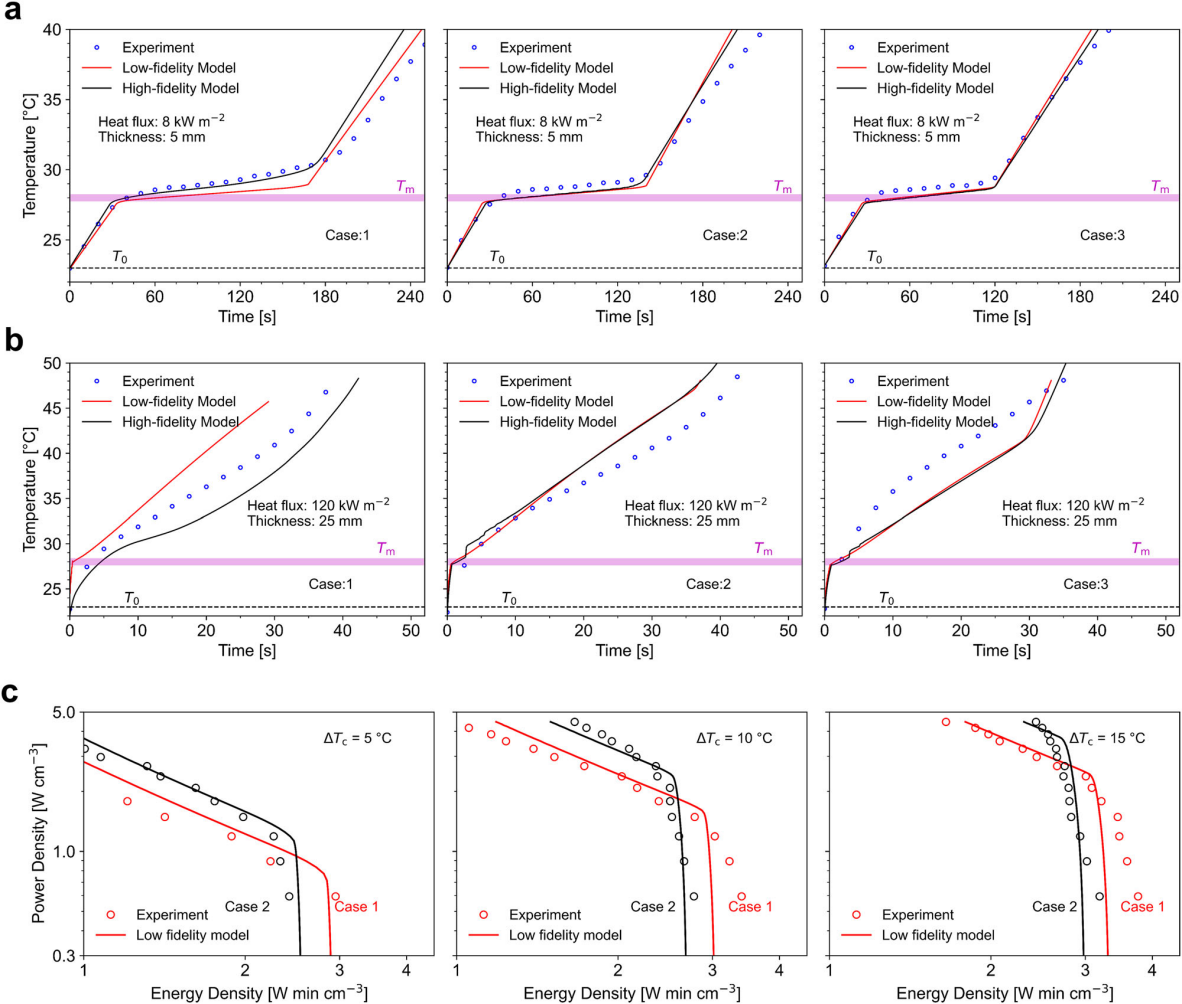
Comparison of the numerical models with experiments. **a** Comparison of transient temperature for high fidelity, low fidelity, and experimental test in three cases at heat flux of 8  kW m^−2^ and thickness of 5 mm, **b** comparison of transient temperature for high fidelity, low fidelity, and experimental test in three cases at heat flux of 120 kW m^−2^ and thickness of 25 mm, **c** comparison of the low fidelity model with the experimental data on the thermal Ragone plot at three cutoff temperatures at thickness of 25 mm.

It can be observed that at low heat flux, as shown in Fig. 5a, generated at heat flux of 8 kW m^−2^ and buffer thickness of 5 mm, both models can predict the transient temperature very well compared to the experimental result, especially in cases 1 and 2, with a mean absolute error between both models and the experiment is around 1.5%. However, as the fin gap width is increased, the high-fidelity model is more accurate than the low-fidelity model for case 3, with the mean absolute error of high-fidelity and low-fidelity models close to 0.9% and 2.2%, respectively, compared to the experiment. Even though there is overall more accuracy in the high-fidelity model than the low-fidelity model, the mean absolute error in the low-fidelity model is still acceptable. The low-fidelity model is relatively much less time-consuming and more economical without compromising the predicted results, as long as the described assumptions are legitimate. The low-fidelity model was approximately 100 times faster than the high-fidelity model for a penalty of only 1.3% in accuracy. At higher heat flux, both models resulted in increased inaccuracy over the experimental results than at low heat fluxes, as depicted in Fig. 5b obtained at a heat flux of 120 kW m^−2^ and a buffer thickness of 25  mm. The mean absolute error at higher heat flux was found to be within 5% for both models; these models are within an acceptable error range. Here, both models were found to be acceptable to be used for the thermal Ragone framework. However, the low-fidelity model can be regarded as a more effective tool considering the accuracy and computational time that can aid in optimizing a PCM-filler thermal buffer.

Figure 5c shows the comparison between power density and energy density through thermal Ragone plots based on experiments on fin design cases and the low-fidelity numerical model at different cutoff temperatures and a fixed thickness of 25 mm. The results show a good match on the thermal Ragone plots for the experimental data and low-fidelity model, with the mean absolute error within 5%.

## Results and discussions

Previously, the Pareto front of maximized energy density for high thermal conductivity fillers and organic PCM is discussed, along with how it compares to pure high thermal conductivity PCMs, such as gallium and field’s metal. Then, we observed that the thermal Ragone curve can be altered by the thickness or aspect ratio and the cutoff temperature. To proceed with this framework of maximizing the energy density, the Pareto front for various variables should be generalized. Heat flux, thickness, and cutoff temperature are some of the variables that could be taken into account in generalization to maximize the energy density. The low-fidelity numerical model is utilized here to obtain the generalized results over a range of heat flux, thickness, and cutoff temperature for different organic PCMs and filler materials. As the results in this section are based on a low-fidelity numerical model, it is worth highlighting the assumptions considered, which are stated as:

- The phase change in the PCM is considered conduction-dominated, and buoyancy-driven natural convection is neglected.
- The composite of PCM and filler material is treated as a homogeneous medium with the effective thermophysical properties.
- The model solves a single energy equation for the effective medium of the composite; hence, the effect of filler morphology is not taken into account.

### Maximizing accessed energy density

The major component of TES in PCM is in the form of latent heat. However, there is some sensible energy storage in the PCM and filler material as well. Under a fixed volume of the composite, the latent energy storage capacity decreases if the addition of filler is increased (ϕ is reduced); in contrast, the sensible energy storage increases as the metal fillers used in this study (copper and aluminum) have higher heat capacity than that of organic PCMs. Intuitively, an extremely low PCM volume fraction is not desired because latent energy is the major contribution to TES. Nevertheless, we analyzed the time scales associated with latent and sensible TES to understand the regime of PCM volume fraction where energy storage is dominated by melting over sensible heating. The melting (latent heating) and sensible heating time scales can be expressed as follows:

1 $${\tau }_{{{\rm{melt}}}}=\,\frac{\phi \rho \Delta H}{q^{\prime\prime} /l}\,$$

2 $${\tau }_{{{\rm{sens}}}}=\,\frac{{C}_{{{\rm{eff}}}}\,\Delta {T}_{s}}{q^{\prime\prime} /l}$$

where, ${C}_{{{\rm{eff}}}}$ is the effective heat capacity of the PCM-metal filler composite medium, which is calculated as the volume-averaged heat capacity of the individual components and can be written as:

3 $${C}_{{{\rm{eff}}}}\,=\,{\phi \cdot C}_{{{\rm{p}}}}\,+\,{\left(1-\phi \right)\cdot C}_{{{\rm{m}}}}$$

**Fig. 6:**
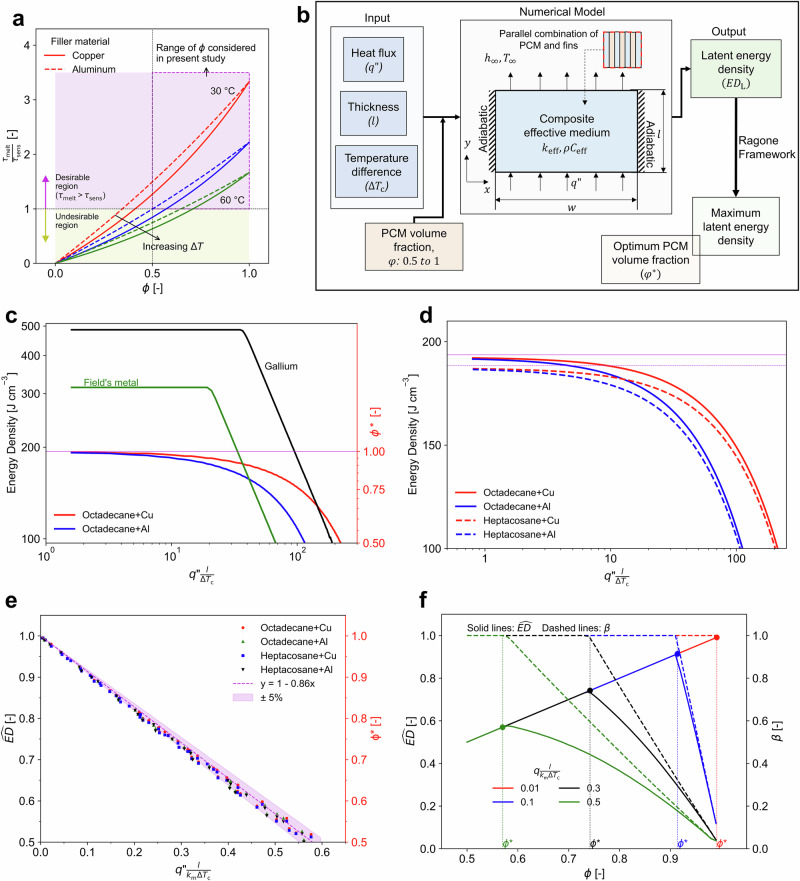
Generalization of the optimized design. **a** Melting and sensible heating time scales, **b** flow chart of maximizing energy density, **c** generalizing the effect of heat flux, thicknesses and cutoff temperature, **d** thermal Ragone curves for different PCM-filler combinations, **e** generalized results based on normalized energy density and input variables, **f** normalized energy density and PCM utilization factor vs. PCM volume fraction, highlighting the optimum points. Note: all the data used to generate these plots are obtained using the low-fidelity model.

As depicted in Fig. 6a, the melting time scale dominates over the sensible heating time scale in the range of PCM volume fraction greater than 0.5. In the next steps, we have considered the latent energy storage capability of the PCM-filler composite to determine the optimum PCM volume fraction.

As discussed earlier, the latent energy density depends on the power density or heat flux, the cutoff temperature, and the thickness. With increasing heat flux and increasing thickness, energy density reduces for a particular design of organic PCM and filler. However, energy density increases with increasing cutoff temperature. Therefore, heat flux, cutoff temperature, and thickness are used as the input variables, and latent energy density as the output. For any combination of the input variables, the objective of the optimization is to determine the design of the PCM-filler composite, i.e., PCM volume fraction (ϕ) that maximizes the amount of thermal energy stored in the PCM as latent energy to depict the utilization of PCM until the cutoff temperature is reached. The input variables are given to the numerical model, and the PCM volume fraction is varied in the range of 0.5–1. The optimum PCM volume fraction and maximum energy density are obtained as shown in Fig. 6b. The optimization approach is utilized to run several cases in the range of input variables for three organic PCMs with different melting temperatures to cover a broad range. The chosen organic PCMs were octadecane, eicosane, and heptacosane with melting temperatures of 28, 37, and 59 °C, respectively. Additionally, two commonly used metal fillers, such as copper and aluminum, were also used. The heat flux is varied from 1.6 to 120 kW m^−2^ in steps of 1.6 kW m^−2^. The thickness of the composite was selected to be 2.5, 5, 10, and 25 mm. Here, instead of the cutoff temperature, the difference between the cutoff and melting temperature (${\Delta T}_{{{\rm{c}}}}={T}_{{{\rm{c}}}}-{T}_{{{\rm{m}}}}$) is used as the input variable. This is done to obtain the results generic to all organic PCMs with different melting temperatures. Four cutoff temperature differences were chosen from 5 to 20 °C. The range of the input variables and the selected PCM and filler materials are mentioned in Table 1.

**Table 1:** Input variables and materials used in the present study for generalization

Parameters	Range
Heat flux (*q*″)	1.6–120 kW m^−2^
Thickness (l)	2.5–25 mm
Cutoff temperature difference (${\Delta T}_{{{\rm{c}}}}$)	5, 10, 15, and 20 °C
Phase change materials	Octadecane, eicosane, heptacosane
Thermal conductive fillers	Copper, aluminum

Figure 6c shows the generalization of the results by considering the effects of heat flux, thickness, and cutoff temperature. It is observed that the energy density decreases linearly with an increase in heat flux and thickness; however, it increases with an increase in the cutoff temperature. However, the curves are different for copper and aluminum as the filler material. This is mainly due to the different thermal conductivity of these materials. For example, copper has higher thermal conductivity than aluminum, which aids in accessing higher energy density at lower filler addition under a given combination of heat flux, thickness, and cutoff temperature. It should be noted here that most of the organic PCMs have similar material properties and mainly differ in the melting temperature. However, the latent energy storage capacity of the organic will still be slightly different, meaning different density and latent heat. Therefore, the thermal Ragone curves for different PCM-filler material combinations can be different, as shown in Fig. 6d. For the generalization of the results based on the material properties of PCM and filler, we can consider normalizing the energy density with the volumetric latent heat of the PCM and heat flux, thickness, and cutoff temperature with the thermal conductivity of the filler material. Figure 6e depicts the normalized energy density and the normalized input variables and filler material. The normalized energy density is obtained by dividing the energy density by the volumetric latent heat of the PCM.

4 $$\hat{{\mathrm{ED}}}=\,\frac{{\mathrm{ED}}}{\rho \Delta H}$$

These results are generalized for all the PCM and filler combinations mentioned in Table 1; however, data points for eicosane are not shown in Fig. 6e for better visibility. The results follow a linear fit with a coefficient of determination greater than 0.99. The normalized energy density leads to the maximum energy density that can be accessed under the given conditions of the input variables, as the results are generalized thermal Ragone Pareto front. The normalized energy density is always equal to the optimum PCM volume fraction (${\phi }^{* }$), meaning the optimum case is achieved when all the PCM is melted. However, complete melting also happens if the PCM volume fraction is chosen lower than the optimum, but the energy density will be lower in this case. Therefore, in the process of finding the optimum PCM volume fraction, we seek the maximum value of the PCM volume fraction at which all the available PCM has melted completely, essentially leading to the maximum accessed latent energy density before the cutoff condition is reached. Figure 6f shows the normalized energy density at different PCM volume fractions, and the optimum is highlighted with circular marker at different values of the normalized input variables. Another parameter that needs to be defined here is the PCM utilization factor (β), which is the ratio of normalized energy density to the PCM volume fraction. This term represents the fraction of PCM that underwent phase change over the available PCM. The optimum case would be the maximum value of *ϕ* where *β* is equal to one. Therefore, normalized energy density can be considered equal to the optimum PCM volume fraction.

The expression for optimum PCM volume fraction is obtained as:

5 $${\phi }^{\ast }=1-a\frac{q^{\prime\prime} l}{{k}_{{{\rm{m}}}}\Delta {T}_{{{\rm{c}}}}}$$

Here, ‘a*’* is a fitting constant and was obtained as 0.86.

Equation 5 is similar to the expression for optimum volume fraction reported in an earlier study^12^. However, in that work, they considered quasi-steady state approximation and analytically solved for the optimum design; in contrast, in this study, we used the validated numerical model to get the power and energy density for the thermal Ragone framework. Therefore, even though both studies are maximizing the operation time or minimizing the temperature, i.e., maximizing the energy storage, the approach to reach this objective is completely different. Another assumption made in the present study, as mentioned earlier, is that the effect of thermal conductivity of the PCM is neglected here because the thermal conductivity of organic PCM is around three orders of magnitude lower than the thermal conductivity of fillers. Regardless, both approaches lead to similar results.

The time to reach the cutoff temperature or cutoff time (${\tau }_{{{\rm{c}}}}$) for the optimum design can be obtained from the energy balance. The heat supplied from the heat source is stored in the form of latent heat (${{\mathrm{ED}}}_{{{\rm{L}}}}$) and sensible heat (${{\mathrm{ED}}}_{{{\rm{S}}}}$). In the energy balance (Eq. 6), the term on the left-hand side is energy density supplied by the heat source to the PCM-filler composite. The first and second terms on the right-hand side are the latent heat stored in PCM and the sensible heat stored in the composite. ${\Delta T}_{{{\rm{s}}}}$ is the difference between the average temperature at the cutoff and the initial condition.

6 $$\frac{q^{\prime\prime} A\,{\tau }_{{{\rm{c}}}}}{V}=\rho \Delta H{\phi }^{\ast }+{C}_{{\mathrm{eff}}}\Delta {T}_{{{\rm{s}}}}$$

7 $${\tau }_{{{\rm{c}}}}=\frac{\rho \Delta H{\phi }^{* }+\,{C}_{{\mathrm{eff}}}{\Delta T}_{{{\rm{s}}}}}{q^{\prime\prime} /l}$$

Equation 5 can be used not only to decide the optimum PCM volume fraction for a given set of conditions, but could also aid in choosing the PCM and filler material. In a general use case, the thickness of the composite is constrained due to space limitations. With heat flux and cutoff temperature known beforehand, the optimum PCM volume fraction and time to reach the cutoff temperature can be obtained from Eqs. 5 and 7, respectively. In the case where the time to reach the cutoff temperature is less than the desired operation time, a new optimum can be obtained by reducing the melting point of the PCM (hence the choice of PCM) or increasing the thermal conductivity of the filler (hence the choice of filler material).

### Optimized design based on power pulse duration

**Fig. 7:**
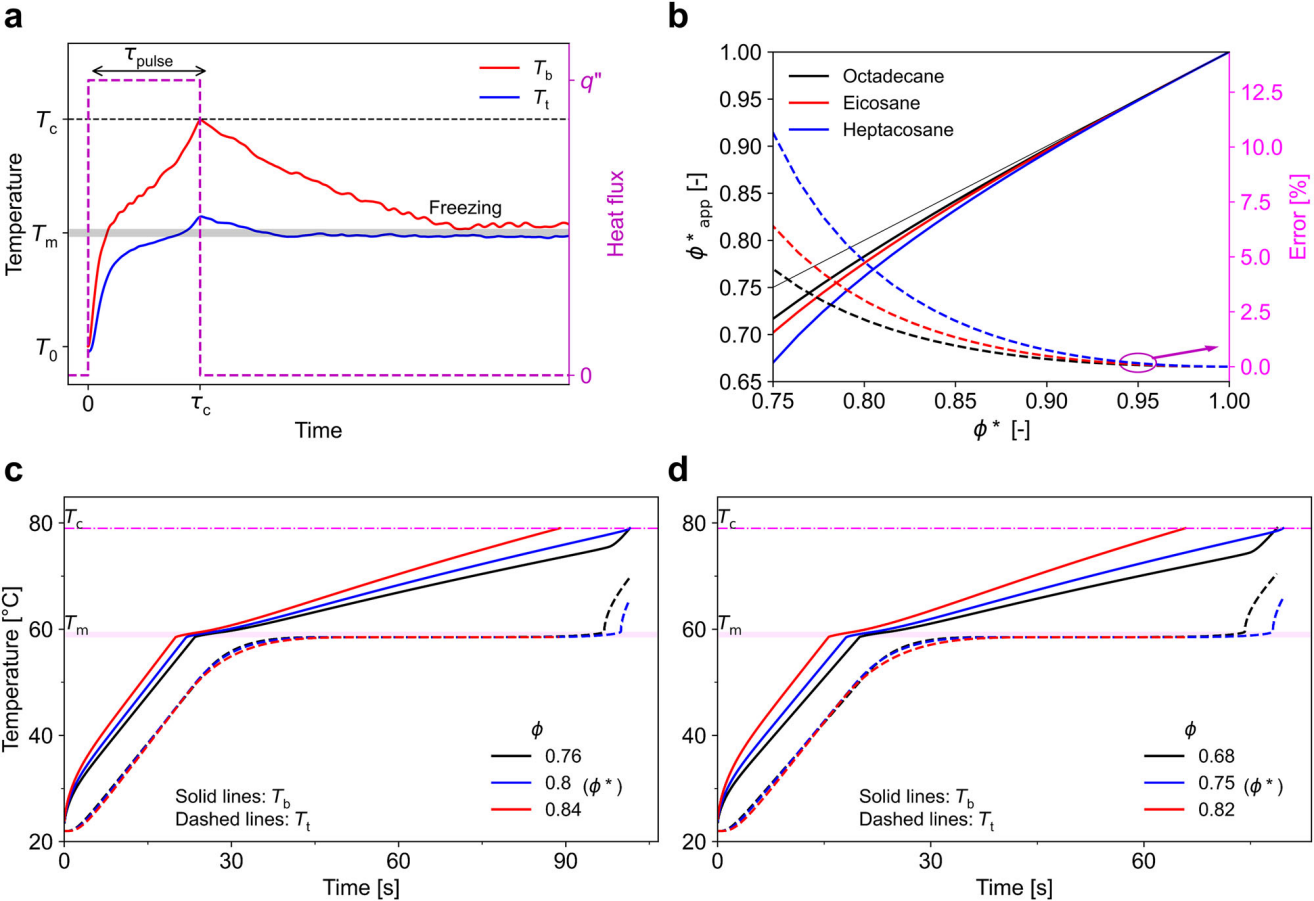
Comparison of approximate and derived results. **a** Transient power pulse for a time duration, **b** derived and approximate optimum PCM volume fraction comparison, **c** transient temperature at and ± 5% of ${\phi }^{* }$ = 0.8, **d** transient temperature at and ± 10% of ${\phi }^{* }$ = 0.75. Note: all the data used to generate these plots are obtained using the low-fidelity model.

In some cases, the time of power pulse input would be the known variable, and the thickness of the PCM-filler composite needs to be obtained to store energy for the given power pulse duration. For a given power pulse ($q^{\prime\prime}$) lasting for a time duration (${\tau }_{{{\rm{pulse}}}}$) as shown in Fig. 7a, we can find the optimum PCM volume fraction by considering the pulse duration as the cutoff time. Therefore, Eq. 5 can be rearranged (see Supplementary Note 3) to obtain the value of ${\phi }^{* }$ that maximizes the energy density under a given power pulse amplitude and time duration. The expression for ${\phi }^{* }$ is obtained as:

8 $${\phi }^{* }=\frac{1}{2}\left[1+\frac{\sqrt{{\left(\rho \Delta H+{C}_{{{\rm{p}}}}{\Delta T}_{{{\rm{s}}}}\right)}^{2}-\frac{4aq^{\prime\prime} {\tau }_{{{\rm{pulse}}}}}{{k}_{{{\rm{m}}}}{\Delta T}_{{{\rm{c}}}}}\left(\rho \Delta H+\left({C}_{{{\rm{p}}}}-{C}_{{{\rm{m}}}}\right){\Delta T}_{{{\rm{s}}}}\right)}{-{C}_{{{\rm{m}}}}\Delta T}_{{{\rm{s}}}}}{\rho \Delta H+\left({C}_{{{\rm{p}}}}-{C}_{{{\rm{m}}}}\right){\Delta T}_{{{\rm{s}}}}}\right]$$

For this optimized design, the required thickness can be calculated using Eq. 5.

In the cases where the melting temperature of PCM is close to the initial temperature and the cutoff temperature is closer to the melting temperature, the sensible energy storage in the metal can be assumed less contributing compared to the latent heat and sensible heat in PCM. Therefore, in those scenarios, the calculation of ${\phi }^{* }$ can be simplified, and we can find an approximate value $({{\phi }^{* }}_{{{\rm{app}}}})$ for ease in calculation, and can be expressed as:

9 $${{\phi }^{* }}_{{{\rm{app}}}}=\frac{1}{2}\left[1+\sqrt{1-\frac{4aq^{\prime\prime} {\tau }_{{{\rm{pulse}}}}}{{k}_{{{\rm{m}}}}{\Delta T}_{{{\rm{c}}}}\cdot \left(\rho \Delta H+{C}_{{{\rm{p}}}}{\Delta T}_{{{\rm{s}}}}\right)}}\,\right]$$

Detailed derivation of Eqs. 8 and 9 is included in the supplementary document (Supplementary Note 3).

Figure 7b compares the prediction of derived ${\phi }^{* }$ and approximate ${\phi }^{* }$ based on Eqs. 8 and 9, respectively. The difference between ${\phi }^{* }$ and$\,{{\phi }^{* }}_{{{\rm{app}}}}$ is very small when ${\phi }^{* }$ is closer to one, however, the error increases as the ${\phi }^{* }$ is decreased. This error increases with the increase in the difference between the melting temperature of the PCM and the initial temperature. Please note that Fig. 7b is generated with copper as the filler material because of its higher sensible heat capacity than aluminum, making it the worst scenario among both materials in terms of error in the approximation. The error percentage was found to be less than 5% and 10% for ${\phi }^{* }$ greater than 0.8 and 0.75, respectively.

To demonstrate the effect of error on the transient temperature during the power pulse, we have analyzed two scenarios. One where ${\phi }^{* }$ is 0.8, alongside ± 5% of error, i.e., ϕ equal to 0.76 and 0.84. Figure 7c depicts the transient temperature for heptacosane and copper composite at three PCM volume fractions at 67.2 kW/m^2^ heat flux. The second scenario is when ${\phi }^{* }$ is 0.75 at heat flux of 84 kW m^−2^, along with ± 10% of error as shown in Fig. 7d. It can be seen in Fig. 7c, d that ${\phi }^{* }$ leads to the maximum time to reach the cutoff temperature. If the ϕ is chosen to be lower than ${\phi }^{* }$, there is a negligibly small decrement in the cutoff time; however, the cutoff time reduces significantly for ϕ chosen to be higher than ${\phi }^{* }$. For $\phi < {\phi }^{* }$, all the available latent energy of the PCM is utilized, whereas in the case of $\phi > {\phi }^{* }$, the PCM does not undergo a complete phase change, and there is still some PCM remaining as a solid when the cutoff temperature is reached on the heated side. It is evident that ${{\phi }^{* }}_{{{\rm{app}}}}$ is always lower than ${\phi }^{* }$ as seen in Fig. 7b. Therefore, the results of the ${{\phi }^{* }}_{{{\rm{app}}}}$ may lead to a conservative design of the PCM-filler composite.

### Cylindrical coordinates

The same set of procedures is also followed for the cylindrical coordinates by utilizing the low-fidelity numerical model. The governing Equations for the low-fidelity model in cylindrical coordinates are detailed in the supplementary document (Supplementary Note 2). The resulting equation for the optimized design was obtained to be:

10 $${\phi }^{* }=1-a\frac{q^{\prime\prime} {r}_{{{\rm{i}}}}{ln}\left(\frac{{r}_{{{\rm{o}}}}}{{r}_{{{\rm{i}}}}}\right)}{{k}_{{{\rm{m}}}}{\Delta T}_{{{\rm{c}}}}}$$

where, ${r}_{{{\rm{i}}}}$ and ${r}_{{{\rm{o}}}}$ are the inner and outer radius of the cylinder.

### Constant temperature boundary condition

As most of the electronic cooling applications are based on heat flux boundary conditions, there are many scenarios where a fixed temperature is set as the boundary condition. The result obtained for constant heat flux boundary conditions applies to constant temperature boundary conditions as well; however, the objective in this case would be to maximize the energy density while maintaining the heat flux below a threshold heat flux (*q*″_th_)^12^. Achieving this objective essentially leads to the same condition at the cutoff temperature as in the case of constant heat flux, where the cutoff temperature in this case would be the same as the constant temperature (${T}_{{{\rm{b}}}}$) maintained at the boundary. Therefore, the results for the optimized design can be written for cartesian (Eq. 11) and cylindrical (Eq. 12) coordinates as:

11 $${\phi }^{* }=1-a\frac{q^{{\prime\prime}}_{{\rm{th}}}\, l}{{k}_{{{\rm{m}}}}\left({T}_{{{\rm{b}}}}-{T}_{{{\rm{m}}}}\right)}$$

12 $${\phi }^{* }=1-a\frac{q^{\prime\prime}_{{\rm{th}}} \,{r}_{{{\rm{i}}}}{ln}\left(\frac{{r}_{{{\rm{o}}}}}{{r}_{{{\rm{i}}}}}\right)}{{k}_{{{\rm{m}}}}\left({T}_{{{\rm{b}}}}-{T}_{{{\rm{m}}}}\right)}$$

In all the expressions for the optimized design, ‘a’ is a fitting constant and equal to 0.86.

All the results obtained in the present study are summarized in Table 2.

**Table 2:** Summarized results for the optimum design

Cartesian coordinate system	Cylindrical coordinate system
	
Constant heat flux (*q*″)	
Objective: maximize (latent energy density, ED_L_) for *T* < *T*_*c*_	
If *q*″ and *l* are known: ${\phi }^{\ast }=1-a\frac{{q}^{\prime\prime} l}{{k}_{{{\rm{m}}}}\Delta {T}_{{{\rm{c}}}}}$ If *q*″ and *τ*_pulse_ are known: Eqs. 8 or 9	${\phi }^{\ast }=1-a\frac{q^{\prime\prime} \,{r}_{{{\rm{i}}}}ln\left(\frac{{r}_{{{\rm{o}}}}}{{r}_{{{\rm{i}}}}}\right)}{{k}_{{{\rm{m}}}}\Delta {T}_{{{\rm{c}}}}}$
Constant surface temperature (*T*_b_)	
Objective: maximize (latent energy density, ED_L_) for *q*″< *q*″_th_	
${\phi }^{* }=1-a\frac{{q^{\prime\prime} }_{{{\rm{th}}}}\,l}{{k}_{{{\rm{m}}}}\left({T}_{{{\rm{b}}}}-{T}_{{{\rm{m}}}}\right)}$	${\phi }^{* }=1-a\frac{{q^{\prime\prime} }_{{{\rm{th}}}}{r}_{{{\rm{i}}}}{ln}\left(\frac{{r}_{{{\rm{o}}}}}{{r}_{{{\rm{i}}}}}\right)}{{k}_{{{\rm{m}}}}\left({T}_{{{\rm{b}}}}-{T}_{{{\rm{m}}}}\right)}$

### Comparison to literature data

Researchers have previously performed several experimental and numerical analyses to determine the optimum volume fraction of PCM in the thermal buffer. In this section, some of that work is discussed in detail and compared to the predicted optimum PCM volume fraction from the present correlation with the results obtained in the literature for planar geometry with a constant heat flux boundary condition. Table 3 details the results of experiments by different authors and how they compare with the present prediction. In a prior study^15^, ${\phi }^{* }$ of 0.92 was reported when tested for five fin-PCM designs across heat fluxes in the range of 3.1–6.2 kW m^−2^. The present prediction found 0.96 and 0.92 as the optimum at 3.1 and 6.2 kW m^−2^, respectively. Similarly, the predicted results were also compared with another experimental study^16^, where four PCM volume fractions were tested in a heat flux range of 2–4 kW m^−2^. They reported the optimum design as ${\phi }^{* }$ = 0.91 for all the heat flux in the range of 1.6–3.2 kW m^−2^. We also compared the present results with the experimental findings at high heat fluxes (48 and 72.5 kW m^−2^)^13^. Overall, the predicted results were found to be very close to the optimum designs reported by other researchers, showing an excellent prediction capability of the present correlations across a wide range of heat flux cutoff temperature and thickness. Hence, aiding the validation of the present results. Nonetheless, it is worth noting that the present results have some limitations, mainly leading from the assumptions considered in the low-fidelity model. The assumption that the composite of organic PCM and metal filler is being treated as a single homogeneous medium with effective properties. This assumption is valid only when the fin spacing is less than a critical length^10^. The model also shows increased error when compared to the experimental results at the upper limit of the heat flux considered in the present study.

**Table 3:** Comparison of the present prediction with the literature-reported/inferred values for planar geometry with constant heat flux boundary

Author	PCM/ filler	*q*″ (kW m^−2^)	$\Delta {T}_{{{\rm{s}}}}$ (°C)	l (mm)	Tested ϕ	Reported/Inferred ${\phi }^{* }$	Predicted ${\phi }^{* }$
Baby and Balaji^16^	Eicosane/ aluminum	2	5.5	25	1, 0.96, 0.91, 0.85	0.91	0.96
		3.2	5.5			0.91	0.94
		4	5.5			0.91	0.92
Saha et al.^15^	Eicosane/ aluminum	3.1	10	25	1, 0.98, 0.92, 0.82, 0.73	0.92	0.97
		6.2	10			0.92	0.93
Tamraparni et al.^13^	Octadecane/ AlSi12alloy	48	17	12	0.8, 0.7, 0.6, 0.5, 0.4, 0.3, 0.2, 0.1	0.6	0.64
		48	22			0.7	0.72
		48	27			0.8	0.77
		72.5	18			0.5	0.48
		72.5	22			0.6	0.57

While prior work has largely treated the analogy of electrochemical and TES as a descriptive metric, the present study establishes it as a design-oriented tool, enabling generalized correlations for optimal composite design across wide range of operating conditions. As a result, this work leverages existing framework to determine the optimum composite design.

## Conclusions

This work leveraged the Ragone framework used in electrochemical energy storage system to optimize composite PCM-based TES systems. Correlations to predict the optimum PCM volume fraction in organic PCM-metal filler composites are proposed. By incorporating key factors, such as operating boundary conditions, geometric configuration, and cutoff temperature, the relationship of the optimum PCM volume fraction on these variables offers a comprehensive yet accessible approach for PCM-based composite design. The validation of this model against in-house experiments and verification of optimum results with findings in the literature demonstrates its potential to reduce the dependency on future experimentation and simulations to make design decisions. The proposed approach can be a generalizable design tool for TES and electronics cooling applications. Future research should focus on expanding the model to incorporate additional parameters with an intermittent operation scenario with a solidification cycle. Furthermore, these models can be incorporated with artificial intelligence to aid in developing a user-friendly, quick decision tool for PCM TES applications.

## Supplementary information

Supplementary_information

## Data Availability

All the data used in this study are available at 10.6084/m9.figshare.31366489.

## Code availability

Code can be provided by the corresponding author upon reasonable request.
