# Supplementary material for: Predictive correlation of optimum phase change material for thermal energy storage
Source: Commun Eng. 2026 Apr 9;5:143. doi: 10.1038/s44172-026-00655-y (PMC13427826; doi:10.1038/s44172-026-00655-y)
Supplement: Supplementary file 1 — Supplementary_information [file 44172_2026_655_MOESM1_ESM.pdf]

Supplementary Information

Predictive Correlation of Optimum Phase Change Material for Thermal Energy Storage

Ayushman Singh[1], Srikanth Rangarajan[2]*, Bahgat Sammakia[1]

[1]Department of Mechanical Engineering, Binghamton University, Binghamton, New York, United States

[2]School of Systems Science and Industrial Engineering, Binghamton University, Binghamton, New York, United States

*Corresponding author email: srangar@binghamton.edu

Supplementary Table 1 Dimensions and PCM volume fraction of three cases tested

Case	t_f (mm)	w_f (mm)	ϕ (-)	Number of fins
1	0.25	1	0.76	19
2	0.25	0.5	0.64	32
3	0.25	0.25	0.49	49

Supplementary Table 2 Material properties of the phase change materials used

Material	Thermal conductivity (W m^{-1} K^{-1})	Density (g cm^{-3})	Specific heat (J g^{-1} K^{-1})	Latent heat of fusion (J g^{-1})	Melting point (°C)
Octadecane	0.36 (s) / 0.15 (l)	0.814	2.14	244	28
Eicosane	0.15	0.778	2.21	247	37
Heptacosane	0.21	0.802	1.92	235	59
Gallium	40.6 (s) / 29.4 (l)	5.91	0.37	80.1	29.8
Field's metal	19	7.9	0.28	39.8	60

Supplementary Note 1: High fidelity model

A two-dimensional computational domain is used to solve the heat transfer in fins and PCM, as well as the fluid flow of molten PCM. This model is developed using the commercially available solver Ansys Fluent 2022 R2 [1]. The high-fidelity model considers the solution of the energy equation in the fins and PCM with the fluid flow in the molten PCM driven by buoyancy. The enthalpy porosity technique is used to solve the phase change process in PCM.

The energy equation for the PCM domain can be expressed as:

$$\rho C_{\mathrm{p}}\left[\frac{\partial T}{\partial t}+\left(u\frac{\partial T}{\partial x}+v\frac{\partial T}{\partial y}\right)\right]= k_{\mathrm{pcm}}\left(\frac{\partial^2 T}{\partial x^2}+\frac{\partial^2 T}{\partial y^2}\right)-\rho\Delta H\frac{\partial \gamma}{\partial t} \quad (1)$$

The energy equation for the fin domain can be expressed as:

$$\rho C_{\mathrm{p}} \frac{\partial T}{\partial t} = k_{\mathrm{fin}} \left(\frac{\partial^2 T}{\partial x^2} + \frac{\partial^2 T}{\partial y^2} \right) \quad 2$$

where ΔH is the latent heat of the PCM, ρC_{p} is the heat capacity, and k is the thermal conductivity.

The continuity equation for the molten PCM can be expressed as:

$$\frac{\partial \rho}{\partial t} + \frac{\partial \rho u}{\partial x} + \frac{\partial \rho v}{\partial y} = 0 \quad 3$$

where ρ is the fluid density; t is time; and u and v are velocities in the x *and* y directions, respectively.

Boussinesq approximation was used where density is defined based on the temperature and thermal expansion coefficient (β).

$$\rho = \rho_{\mathrm{ref}}[1 - \beta(T - T_{\mathrm{ref}})] \quad 4$$

The momentum equations for the molten PCM can be expressed as:

$$\rho \left(\frac{\partial u}{\partial t} + u \frac{\partial u}{\partial x} + v \frac{\partial u}{\partial y} \right) = \mu \left(\frac{\partial^2 u}{\partial x^2} + \frac{\partial^2 u}{\partial y^2} \right) - \frac{\partial p}{\partial x} + S_{\mathrm{x}} \quad 5$$

$$\rho \left(\frac{\partial v}{\partial t} + u \frac{\partial v}{\partial x} + v \frac{\partial v}{\partial y} \right) = \mu \left(\frac{\partial^2 v}{\partial x^2} + \frac{\partial^2 v}{\partial y^2} \right) - \frac{\partial p}{\partial y} + S_{\mathrm{y}} \quad 6$$

where S_x and S_y are momentum source terms in x and y directions, respectively, and is given as:

$$S_{\mathrm{x}} = \frac{(1-\gamma)^2}{(\gamma^3 + \varepsilon)} A_{\mathrm{m}} u \quad 7$$

$$S_{\mathrm{y}} = \frac{(1-\gamma)^2}{(\gamma^3 + \varepsilon)} A_{\mathrm{m}} v - \rho g \quad 8$$

The source terms are derived based on the enthalpy porosity scheme [2], where γ is the liquid fraction of PCM, i.e., γ = 0 (solid) and $\gamma = 1$ (liquid). γ is defined based on the temperature as follows:

$$\gamma = \begin{cases} 0; if\ T < T_{\mathrm{s}} \\ 1; if\ T > T_{\mathrm{l}} \\ \dfrac{T - T_{\mathrm{s}}}{T_{\mathrm{l}} - T_{\mathrm{s}}}; if\ T_{\mathrm{s}} < T < T_{\mathrm{l}} \end{cases} \quad 9$$

where T_{s} and T_{l} are the solidus and liquidus temperatures of PCM, respectively.

In the source terms, ε is a small number equal to 0.001 to avoid the term tending to infinity as γ tends to zero. Also, A_{m} is the mushy zone constant for the mixture of solid and liquid PCM and signifies the inverse of the permeability to mimic Carman-Kozeny equations. In this study, A_{m} is taken as 10^8 kg m^{-3} s^{-1} based on the best agreement of experimental and numerical model results [3]. The second term in the source term in the y-direction represents the buoyancy-driven flow of liquid PCM.

Supplementary Note 2: Low fidelity model

This model is developed by generating code using MATLAB R2024a [4]. The low-fidelity model considers the solution of a single energy equation for the homogeneous medium of the composite of PCM and metal fillers. Supplementary Fig. 1 shows the schematic describing the difference between the high and low fidelity models. The low-fidelity model is based on the following assumptions:

1. Heat transfer is conduction-dominated, and convection is ignored.
2. The composite of fins and PCM is treated as a single homogeneous medium with effective thermophysical properties.
3. Fluid flow in the molten PCM is ignored.

Cartesian Coordinates

The energy equation for the effective medium of the PCM and fin in cartesian coordinates can be expressed as:

$$\left(\rho C_{\mathrm{p}}\right)_{\mathrm{eff}} \frac{\partial T}{\partial t} = \left(k_{\mathrm{eff}} \frac{\partial^2 T}{\partial y^2}\right) - \phi \rho \Delta H \frac{\partial \gamma}{\partial t} \tag{10}$$

The effective thermal properties of the PCM-fins composite are calculated based on the volume averaging and are expressed as:

Effective heat capacity and thermal conductivity:

$$(\rho C)_{\mathrm{eff}} = \phi \cdot (\rho C)_{\mathrm{pcm}} + (1-\phi) \cdot (\rho C)_{\mathrm{fin}} \tag{11}$$

$$k_{\mathrm{eff}} = \phi \cdot k_{\mathrm{pcm}} + (1-\phi) \cdot k_{\mathrm{fin}} \tag{12}$$

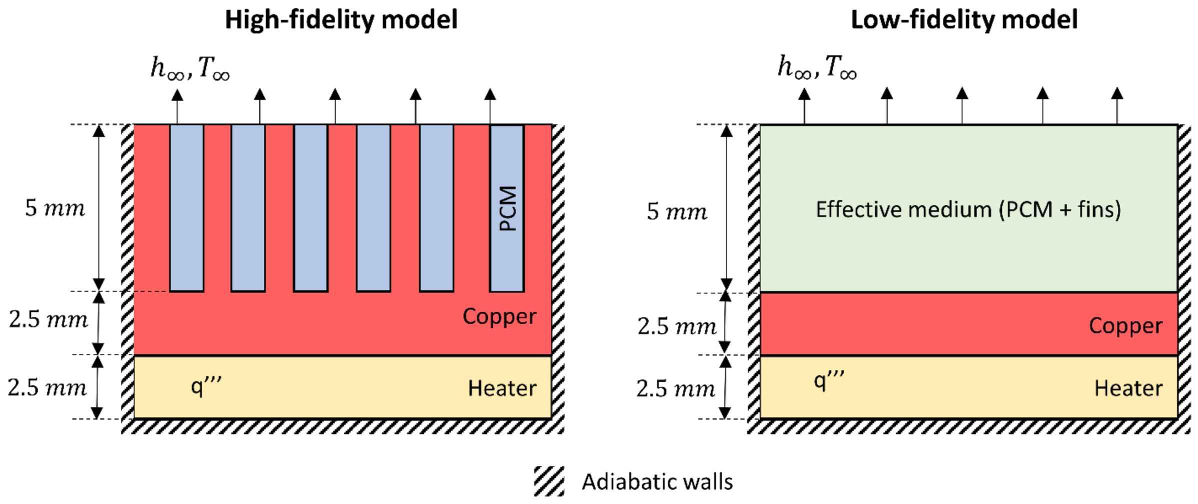

Supplementary Fig. 1 Schematic showing the high-fidelity and low-fidelity models

Cylindrical Coordinates

The energy equation for the effective medium of the PCM and fin in cartesian coordinates can be expressed as:

$$(\rho C_{\mathrm{p}})_{\mathrm{eff}} \frac{\partial T}{\partial t} = \frac{1}{r} \frac{\partial}{\partial r}\left(k_{\mathrm{eff}} \frac{\partial T}{\partial r}\right) - \phi \rho \Delta H \frac{\partial \gamma}{\partial t} \tag{13}$$

For equations 10 and 13, γ is the same as written in equation 9.

The schematic of the computational domain in cartesian and cylindrical coordinates is shown in Supplementary Fig. 2.

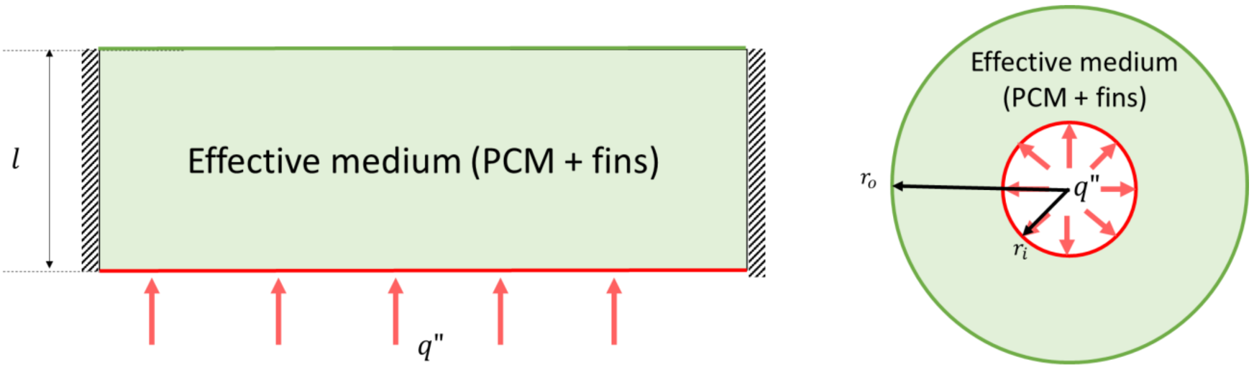

Supplementary Fig. 2 Schematic of the low fidelity model in cartesian (left) and cylindrical coordinates (right)

Supplementary Note 3: Derivation of optimized PCM volume fraction based on power pulse duration

The expression (Equation 5 in the paper) for optimized PCM volume fraction in Cartesian coordinates based on space constraint was obtained as:

$$\phi^* = \left(1 - a \frac{q"l}{k_{\mathrm{m}} \Delta T_{\mathrm{c}}}\right) \tag{14}$$

We can then write the energy balance for this optimized composite as:

$$\frac{q"A\,\tau_{\mathrm{c}}}{V} = \rho \Delta H \phi^* + C_{\mathrm{eff}} \Delta T_{\mathrm{s}} \tag{15}$$

The time to reach the cutoff temperature (cutoff time) can be determined from the above equation in the following form:

$$\tau_{\mathrm{c}} = \frac{\rho \Delta H \phi^* + C_{\mathrm{eff}} \Delta T_{\mathrm{s}}}{q"/l} \tag{16}$$

The thickness of the composite required to achieve this cutoff time while maintaining the optimized design will be:

$$l = \frac{q"\tau_{\mathrm{c}}}{\rho \Delta H \phi^* + C_{\mathrm{eff}} \Delta T_{\mathrm{s}}} \tag{17}$$

For the use case scenario where the power pulse time and heat flux (amplitude of power pulse per unit area) are known, we can put the Equation 17 for $'l'$ into Equation 14 and replace the cutoff time with the power pulse time.

$$\varphi^* = 1 - a \frac{q"}{k_{\mathrm{m}} \Delta T_{\mathrm{c}}} \cdot \frac{q"\tau_{\mathrm{pulse}}}{\rho \Delta H \phi^* + C_{\mathrm{eff}} \Delta T_{\mathrm{s}}} \tag{18}$$

We know that effective heat capacity is volume averaged heat capacity of the PCM and filler and is written as:

$$C_{\mathrm{eff}} = \phi^* \cdot C_{\mathrm{p}} + (1 - \phi^*) \cdot C_{\mathrm{m}} \qquad (19)$$

Equation 18 can then be rewritten as:

$$\phi^* = 1 - a \frac{q''}{k_{\mathrm{m}}\, \Delta T_{\mathrm{c}}} \cdot \frac{q'' \tau_{\mathrm{pulse}}}{\rho \Delta H \phi^* + \phi^* (C_{\mathrm{p}} - C_{\mathrm{m}}) \Delta T_{\mathrm{s}} + C_{\mathrm{m}} \Delta T_{\mathrm{s}}}$$

After further manipulations, we can get a quadratic equation of the form $A\varphi^{*2} + B\varphi^* + C = 0$:

$$\left[\rho \Delta H + (C_{\mathrm{p}} - C_{\mathrm{m}})\Delta T_{\mathrm{s}}\right]\phi^{*2} - \left[\rho \Delta H + (C_{\mathrm{p}} - 2C_{\mathrm{m}})\Delta T_{\mathrm{s}}\right]\phi^* + a \frac{q''^2 \tau_{\mathrm{pulse}}}{k_{\mathrm{m}} \Delta T_{\mathrm{c}}} - C_{\mathrm{m}} \Delta T_{\mathrm{s}} = 0$$

Here, $A = \left[\rho \Delta H + (C_{\mathrm{p}} - C_{\mathrm{m}})\Delta T_{\mathrm{s}}\right]$; $B = -\left[\rho \Delta H + (C_{\mathrm{p}} - 2C_{\mathrm{m}})\Delta T_{\mathrm{s}}\right]$ and $C = a \frac{q''^2 \tau_{\mathrm{pulse}}}{k_{\mathrm{m}} \Delta T_{\mathrm{c}}} - C_{\mathrm{m}} \Delta T_{\mathrm{s}}$

The solution to this quadratic equation is: $\varphi^* = \frac{-B \pm \sqrt{B^2 - 4AC}}{2A}$ which can be written as:

$$\phi^* = \frac{\left[\rho \Delta H + (C_{\mathrm{p}} - 2C_{\mathrm{m}})\Delta T_{\mathrm{s}}\right] \pm \sqrt{\left[\rho \Delta H + (C_{\mathrm{p}} - 2C_{\mathrm{m}})\Delta T_{\mathrm{s}}\right]^2 - 4\left[\rho \Delta H + (C_{\mathrm{p}} - C_{\mathrm{m}})\Delta T_{\mathrm{s}}\right]\left[a \frac{q''^2\, \tau_{\mathrm{pulse}}}{k_{\mathrm{m}} \Delta T_{\mathrm{c}}} - C_{\mathrm{m}} \Delta T_{\mathrm{s}}\right]}}{2\left[\rho \Delta H + (C_{\mathrm{p}} - C_{\mathrm{m}})\Delta T_{\mathrm{s}}\right]}$$

After further manipulations, the solution is found to be:

$$\phi^* = \frac{1}{2}\left[1 - \frac{C_{\mathrm{m}} \Delta T_{\mathrm{s}}}{\rho \Delta H + \left(C_{\mathrm{p}} - C_{\mathrm{m}}\right)\Delta T_{\mathrm{s}}} \pm \frac{\sqrt{\left(\rho \Delta H + C_{\mathrm{p}} \Delta T_{\mathrm{s}}\right)^2 - \frac{4 a q''^2 \tau_{\mathrm{pulse}}}{k_{\mathrm{m}} \Delta T_{\mathrm{c}}}\left(\rho \Delta H + \left(C_{\mathrm{p}} - C_{\mathrm{m}}\right)\Delta T_{\mathrm{s}}\right)}}{\rho \Delta H + \left(C_{\mathrm{p}} - C_{\mathrm{m}}\right)\Delta T_{\mathrm{s}}}\right]$$

Please note that, for the solution of φ^* to be greater than 0.5, a positive sign should be considered in the above equation. Therefore, the final solution (Equation 8 in the paper) can be written as:

$$\phi^* = \frac{1}{2}\left[1 + \frac{\sqrt{\left(\rho \Delta H + C_{\mathrm{p}} \Delta T_{\mathrm{s}}\right)^2 - \frac{4 a q''^2 \tau_{\mathrm{pulse}}}{k_{\mathrm{m}} \Delta T_{\mathrm{c}}}\left(\rho \Delta H + \left(C_{\mathrm{p}} - C_{\mathrm{m}}\right)\Delta T_{\mathrm{s}}\right)} - C_m \Delta T_s}{\rho \Delta H + \left(C_{\mathrm{p}} - C_{\mathrm{m}}\right)\Delta T_{\mathrm{s}}}\right] \qquad (20)$$

Furthermore, the approximate value of the optimized PCM volume fraction of the PCM can be obtained based on the assumption that the sensible energy storage in the filler material is negligible compared to that of the PCM. With this assumption, the effective sensible heat capacity can be approximated as:

$$C_{\mathrm{eff}} \approx \phi^* \cdot C_{\mathrm{p}}$$

$$\phi^* = 1 - a \frac{q''}{k_{\mathrm{m}} \Delta T_{\mathrm{c}}} \cdot \frac{q'' \tau_{\mathrm{pulse}}}{\phi^* (\rho \Delta H + C_{\mathrm{p}} \Delta T_{\mathrm{s}})}$$

$$\phi^{*2} - \phi^* + a \frac{q''^2 \tau_{pulse}}{k_{\mathrm{m}} \Delta T_{\mathrm{c}} \left(\rho \Delta H + C_{\mathrm{p}} \Delta T_{\mathrm{s}}\right)} = 0$$

The solution for the quadratic equation in this case is:

$$\phi^*_{\mathrm{app}} = \frac{1}{2}\left[1 \pm \sqrt{1 - \frac{4aq''^2\tau_{\mathrm{pulse}}}{k_{\mathrm{m}}\Delta T_{\mathrm{c}} \cdot \left(\rho\Delta H + C_{\mathrm{p}}\Delta T_{\mathrm{s}}\right)}}\right]$$

Again, for the solution of φ^*_{app} to be greater than 0.5, positive sign should be considered. Hence, the approximate solution (Equation 9 in the paper) can be written as:

$$\phi^*_{\mathrm{app}} = \frac{1}{2}\left[1 + \sqrt{1 - \frac{4aq''^2\tau_{\mathrm{pulse}}}{k_{\mathrm{m}}\Delta T_{\mathrm{c}} \cdot \left(\rho\Delta H + C_{\mathrm{p}}\Delta T_{\mathrm{s}}\right)}}\right] \quad 21$$

Supplementary References

1. Ansys Fluent | Fluid Simulation Software. https://www.ansys.com/products/fluids/ansys-fluent.
2. Voller, V. R. & Prakash, C. A fixed grid numerical modelling methodology for convection-diffusion mushy region phase-change problems. *International Journal of Heat and Mass Transfer* **30**, 1709–1719 (1987).
3. Liu, Z., Yao, Y. & Wu, H. Numerical modeling for solid–liquid phase change phenomena in porous media: Shell-and-tube type latent heat thermal energy storage. *Applied Energy* **112**, 1222–1232 (2013).
4. MATLAB. https://www.mathworks.com/products/matlab.html.
